# Identifying Potential Markers for Monitoring Progression to Ovarian Cancer Using Plasma Label-free Proteomics

**DOI:** 10.7150/jca.50733

**Published:** 2021-01-15

**Authors:** Wenjie Wang, Hongyu Xie, Bairong Xia, Liuchao Zhang, Yan Hou, Kang Li

**Affiliations:** 1 Department of Biostatistics, School of Public Health, Harbin Medical University, Harbin 150086, China.; 2 Department of Gynecology Oncology, Harbin Medical University Cancer Hospital, Harbin 150000, China.

**Keywords:** ovarian cancer, proteomics, diagnosis, progression, CRP

## Abstract

**Background:** Cancer antigen 125 (CA125) is considered to have high sensitivity but poor specificity for ovarian cancer. New biomarkers utilized to early detect and monitor the progression of ovarian cancer patients are critically needed.

**Methods:** A total of 80 patients including 16 early stage, and matched with 17 late stage, 23 benign ovarian tumor (BOT) and 24 uterine fibroid (UF) patients were utilized to perform plasma proteomics analysis using isobaric tag for relative and absolute quantitation (iTRAQ) method to identify differential diagnostic proteins of ovarian cancer patients. A validation set of 9 early stage, 11 late stage, 17 BOT and 16 UF collected by an independent cohort of samples with the same matching principles was examined to confirm the expressed levels of differential expression proteins by ELISA analysis.

**Results:** CRP and ARHGEF 11 were identified as potential diagnostic biomarkers of ovarian cancer. Results of area under the curve (AUC) analysis suggested that combination of diagnostic proteins and CA125 achieved a much higher diagnostic accuracy compared with CA125 alone (AUC values: 0.98 versus 0.80), especially improved the specificity (0.97 versus 0.77). In addition, elevated plasma CRP levels were associated with increased risk of ovarian cancer.

**Conclusions:** Current study found that plasma protein CRP was an indicator for monitoring the progression of ovarian cancer. Combination of plasma protein biomarkers with CA125 could be utilized to early diagnose of ovarian cancer patients.

## Introduction

Epithelial ovarian cancer (EOC) remains the deadliest gynecologic malignancy around the world 1, 2. Although the 5-year survival rate for patients diagnosed at an early stage (I and II) is over 90%, this decreases to 30% in advanced diagnoses (III and IV) 3. Unfortunately, most patients suffering from EOC are diagnosed at advanced stages because no reliable and accurate screening test exist currently and with the characteristic of asymptomatic during its early stage. At present, standard methods for screening ovarian cancer are ultrasonography and the serum carbohydrate antigen 125 (CA125) 4, 5. CA125 remains the best serum tumor marker for ovarian cancer, nevertheless increased serum CA125 levels may give false positive results in benign gynecological conditions and other cancers 5-7. Thus, identifying biomarkers with higher sensitivity and specificity for early detection and dynamic disease monitoring remains a major unmet clinical need for ovarian cancer.

Cancer is a genetic defect that drives abnormal cellular proliferation, and the alteration observed at the genome levels are manifested at the protein level, because the proteins drive the abnormal phenotype 8, 9. Proteomics is a powerful tool that monitors and detects the changes in protein expression in cells. Some studies demonstrated that dysregulated protein expressed was associated with cancers. Pappa et al. identified differentially expressed proteins between the normal and cervical cancer lines using 2-dimensional electrophoresis, and the differentially expressed proteins were further validated by MALDI-TOF and western blot analysis, 113 proteins were found to dysregulated in cancer lines at last compared with normal control 10. Matthew et al. conducted a prospective study to identify 90 differentially expression proteins between ovarian cancer and controls based isobaric tag for relative and absolute quantitation (iTRAQ) method, followed by ELISA analysis, and finally only protein Z was recognized as a novel biomarker for early detection for ovarian cancer. However, this study just used healthy population as a control sample and has not included samples from patients with benign tumors, which limited the clinical utility 11. Currently, 2-dimensional electrophoresis 12, 2-dimensional difference gel electrophoresis 13, stable isotope labeling with amino acids in cell culture 14, and iTRAQ 15 are the techniques that widely used in identifying cancer protein biomarkers. However, 2-dimensional electrophoresis as a fundamental technique in the protein detection has several disadvantages, such as low sensitivity, poor reproducibility, and insufficient linear range of visualization 16. Several studied have indicated that the iTRAQ technique is a highly sensitive, quantitative, and reproducible method for detection of differentially expressed proteins, which has been widely used in cancer research 17. The application of proteomics technologies to identify novel biomarker would push the boundaries of diagnosis and monitoring the progression of disease.

In this study, we used the iTRAQ approach to examine plasma differential protein expression for diagnosing of ovarian cancer. These differentially expressed proteins were further validated by ELISA detection and identified novel protein biomarkers in monitoring the progression of ovarian cancers. In addition, we further evaluated the specificity, sensitivity and the prediction accuracy of novel proteins alone and together with CA125 for detecting EOC to promote the clinical utility and monitoring progression.

## Methods

### Study population

This study was approved by the Ethics Committee of the Tumor Hospital of Harbin Medical University, and all patients signed informed consents before the study began. Participants who were suffering from metabolic diseases, liver diseases, kidney diseases, or any other cancers were excluded. Patients who were diagnosed with early stage (stage I-II), matched with late stage (stage III-IV), benign ovarian tumor (BOT) and uterine fibroid (UF) and received surgery between August, 2009 and April, 2013, which were matched on age (± 5 years), menopausal status and admission date (± 6 months) by 1: 1: 2: 2.

In the validation phase, we used a separate, independent cohort of samples collected through the Department of Gynecology of Harbin Medical University Tumor Hospital (Harbin, China) between May, 2013 and April, 2015 with the same matching principles (age (± 5 years), menopausal status and admission date (± 6 months)), which were analyzed with ELISA method. Complete demographic and clinical information were collected for all specimens by members of clinical study team.

### Sample collection

Plasma samples were collected from pretreatment primary ovarian cancer patients and benign controls at the Department of Gynecology of Harbin Medical University Tumor Hospital. Fasting venous blood samples were collected using vacuum blood collection tube contained anticoagulant dipotassium EDTA. Plasma was separated by centrifugation at 1,323g for 10 min and the supernatant was stored at -80℃ until further analysis.

### iTRAQ Labeling and Proteomics Detection

Protein was reduced with 5mM dithiothreitol (DTT) at 37℃ for 1h and alkylated with 20mM iodoacetamide (IAA) in the dark for 1h. The proteins were digested using sequencing-grade trypsin (Promega) at a concentration of 1:50 trypsin/protein at 37 ℃ overnight. Using the 8-plex iTRAQ reagent to label the resulting peptide mixture based on the manufacturer's instructions (Applied Biosystems SCIEX). The samples were labeled as follows: early stage sample was tagged with 113, late stage sample with 114, UF and BOT samples with 119 and 121, respectively. Then using SCX chromatography based on the AKTA Purifier system (GE Healthcare) to divide the iTRAQ labeled peptides. The labeled peptide mixtures were reconstructed and acidified with 2 ml buffer A (10 mM KH2PO4 in 25% of ACN, pH 2.7) and loaded onto a 4.6 x 100 mm Polysμlfoethyl column (5 µm, 200 Å, PolyLC Inc, Maryland, U.S.A.). Detection was performed on a Q Exactive (Thermo Fisher Scientific, San Jose, CA) mass spectrometer that was coupled to Ulimate3000. Each fraction (10 ml) was injected for nanoLC-MS/MS analysis 18.

### Sequence Database Searching and Data Analysis

MS/MS spectra were searched using MASCOT engine (Matrix Science, London, UK; version 2.2) against a non-redundant International Protein Index arabidopsis sequence database v3.85 from the European Bioinformatics Institute (http://www.ebi.ac.uk/). For protein identification, the options were as followed: Peptide mass tolerance=20 ppm, MS/MS tolerance=0.1 Da, Enzyme=Trypsin, Missed cleavage=2, Fixed modification: iTRAQ 8 plex (K), iTRAQ 8 plex (N-term), Variable modification: Oxidation (M), Decoy database pattern=Reverse. The MASCOT search results for each SCX elution were further processed using the Proteomics Tools (version 3.05) http://www.proteomics.ac.cn/) 19.

### ELISA validation

Briefly, standards were prepared and samples were diluted as determined by an optimization step performed previously, then 100 µL of these were added to the appropriate wells of the ELISA plate and incubated at 37°C for 2hs. Following aspiration of the samples and standards, 100 µL of biotin conjugated antibody was added and incubated for a further 1 h at 37°C. Wells were washed with the provided Wash Buffer, then HRP-avidin detection reagent was added and incubated at 37°C for 30 minutes (USCN kits) or 1h (Cusabio kits and VWA5B2 My Biosource kit). Detection reagent was then removed and the wells were washed again with Wash Buffer before adding substrate solution and incubation for another 15-30 minutes at 37°C. A stop solution was then added and the optical density of each well was read at 450 nm.

The TSR1 My Biosource kit procedure was different in that 50 µL standard and sample were used and were added to the plate followed by the addition of 100 µL of HRP-conjugate followed by a 60 minute incubation at 37°C. After four washes with the wash buffer, 50 µL each of Chromogen solution A and 50 µL of Chromogen solution B were added to the wells and this mixture was incubated at 37°C for 15 minutes. Finally, 50 µL of stop solution was added prior to the plate being read at 450 nm 20.

### Statistics analysis

For each group studied by MS, there were two technical replicates and two sample replicates, resulting in four ratios for each comparison. Because of the variability observed between replicates, a measure, termed the 'regulation score' [Equation (1)] was used to summarize the magnitude and consistency of differential abundance across multiple derives log2(ratios) 21. Proteins located in the top 40 most consistently regulated, proteins identified with ≥ 2 peptides and significant (p<0.05) were selected as high confidence proteins. High confidence proteins with fold change (≥2 or <0.5) across groups were used for further validating markers. The differences in the intensities of proteins in different groups were compared using the Kruskal-Wallis rank sum test. SNK (q-test) was utilized to compare the difference between every pair of groups. Area under the receiver operating characteristic (AUC) analysis and correct prediction rate were used to evaluate predictive performance.


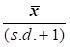
(1)

## Results

### Patient characteristics

**Figure 1:**
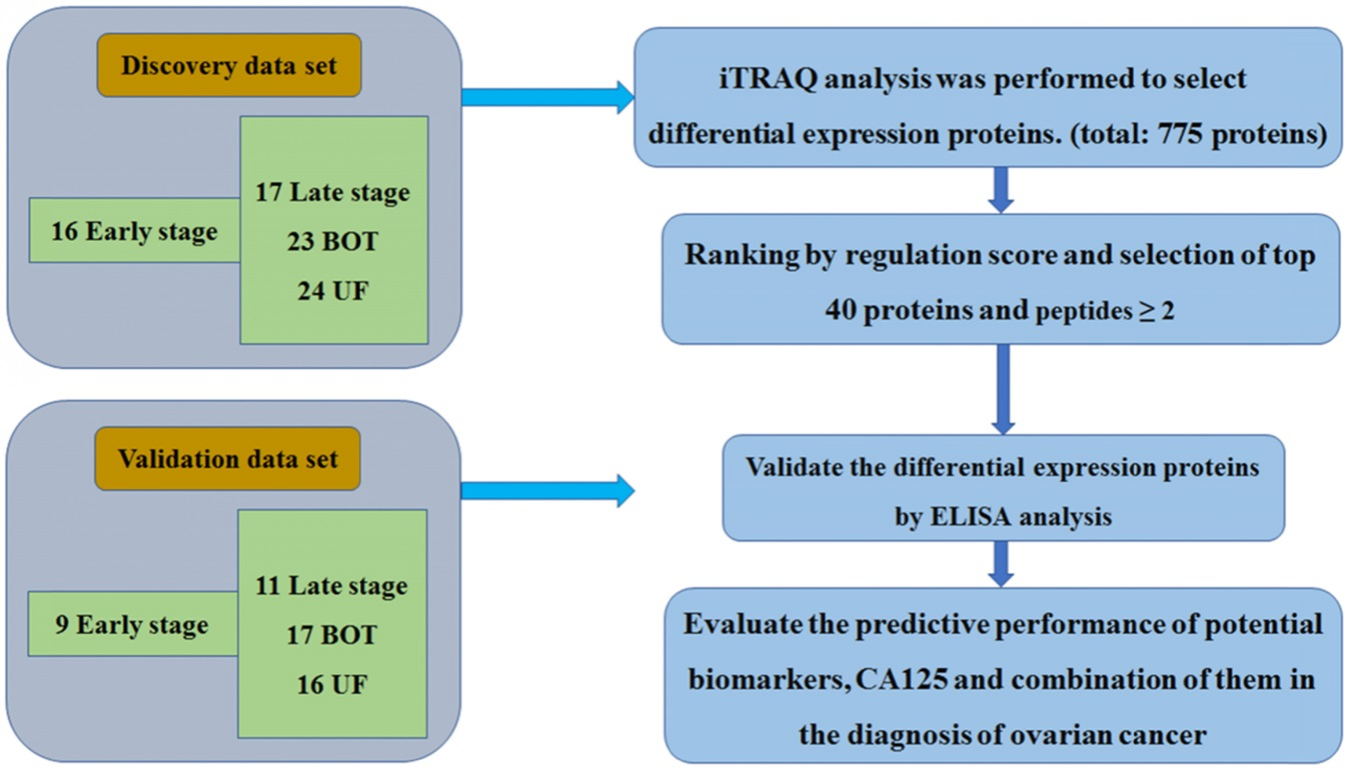
The workflow of this study

Plasma specimens from 80 women were collected for iTRAQ analysis. The breakdown of specimen numbers was as follows: 16 stage I-II, 17 stage III-IV, 23 BOT and 24 UF patients. For the validation set, a separate, independent cohort of samples including 9 stage I-II, 11 stage III-IV, 17 BOT and 16 UF were utilized to further ELISA analysis. The patient characteristics in the discovery set and validation set were listed in Table 1. The specific workflow was displayed in Fig. 1.

### Summary of proteomic analysis of EOC plasma

iTRAQ analysis was performed on the discovery set of paired stage I-II, stage III-IV, BOT and UF plasma samples. Two technical replicates and two sample replicates were performed to determine the reproducibility of the experimental results. A total of 775 proteins were identified and 698 proteins were identified with excellent reproducibility and significant (p<0.05). 214 proteins with ≥ 2 peptides were selected as high confidence proteins. From this total, we shortlisted 40 for further study based on the regulation score mentioned previously. Four high confidence proteins were identified that with fold change ≥2 or <0.5 to differentiate one group from other three groups and identified as differential expression proteins used for further validating markers. Of this four proteins were selected for validation by ELISA, which were summarized in Table 2.

### Comparison with Tissue proteomics

To further validate the consistency of results between the plasma protein and the tissue, we compared these plasma proteomics results with those of ovarian cancer tissue from the Clinical Proteomic Tumor Analysis Consortium (CPTAC) database. CPTAC used iTRAQ in conjunction with offline liquid chromatography fractionation via high-pH reverse-phase liquid chromatography (RPLC) and online RPLC with high-resolution tandem MS to perform proteomics analysis of ovarian cancer tissue and provide protein identification and quantification. In our results, 775 proteins were identified in the current proteomics analysis of plasma, and 426 proteins overlapped between tissue and plasma. Among these common proteins, 387 proteins with late stage and early stage ratios in proteomics analysis of plasma, 319 proteins were secretory protein secreted from cell and had biological function in the extracellular, were divided into four parts. Compared with early stage patients, 69 proteins were up-regulated in both plasma and tissue and 26 proteins were secretory protein (37.68%), 128 proteins were down-regulated in both plasma and tissue and 79 proteins were secretory protein (61.72%), 116 proteins were up-regulated in plasma while down-regulated in tissues and 76 proteins were secretory protein (65.51%), 74 proteins were down-regulated in plasma while up-regulated in tissues and 21 proteins were secretory protein (28.38%). 53.3% of the secretory proteins in the plasma and tissue had the same trend while 51.05% of the secretory proteins in the plasma and tissue had the different trend. The details were provided in the Fig. S1 and Table S1.

### Biomarkers validated by ELISA

**Figure 2:**
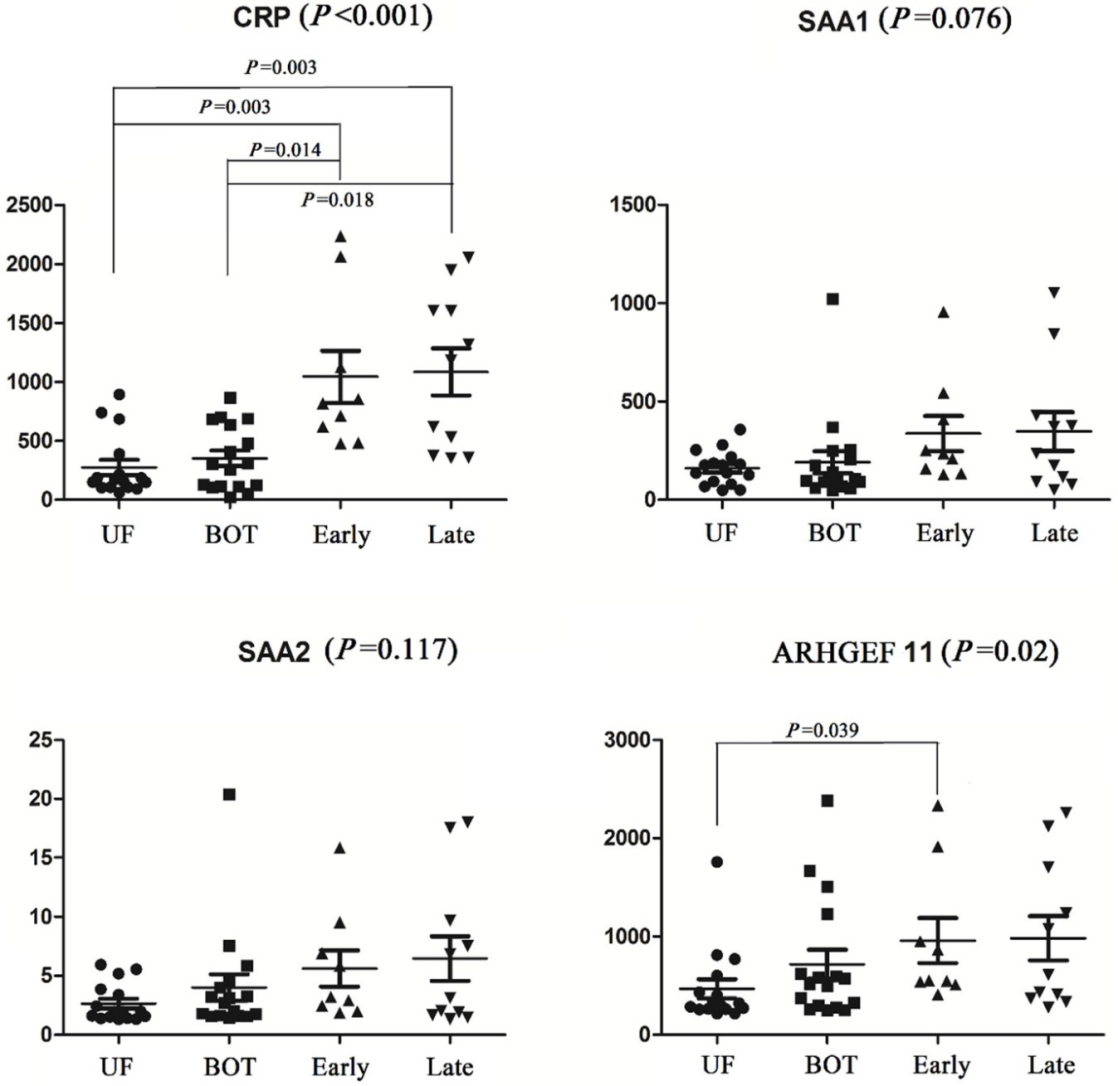
Elisa analysis of differentially expressed proteins identified in the discovery set.

The identified different proteins were validated as ovarian cancer potential biomarkers for progressive disease by ELISA, which were performed on an independent cohort of patients. Kruskal-Wallis analysis of ELISA data demonstrated that CRP and ARHGEF-11 were significantly differentially expressed across the groups with *p* values of <0.001 and 0.02, respectively. SNK analysis showed significant CRP increases in levels in cancer patients and controls (UF vs early stage, *P*=0.003; UF vs late stage, *P*=0.003; BOT vs early stage, *P*=0.014; BOT vs late stage, *P*=0.018), but there were no differences between early stage and late stage of ovarian cancers. The ARHGEF 11 ELISA concentration analysis showed significantly expressed between UF and early stage of ovarian cancer patients. While SAA1 and SAA2 were found not to be significant in abundance across the groups by Kruskal-Wallis analysis (Fig. 2).

### Evaluation of the predictive performance of proteins

**Figure 3:**
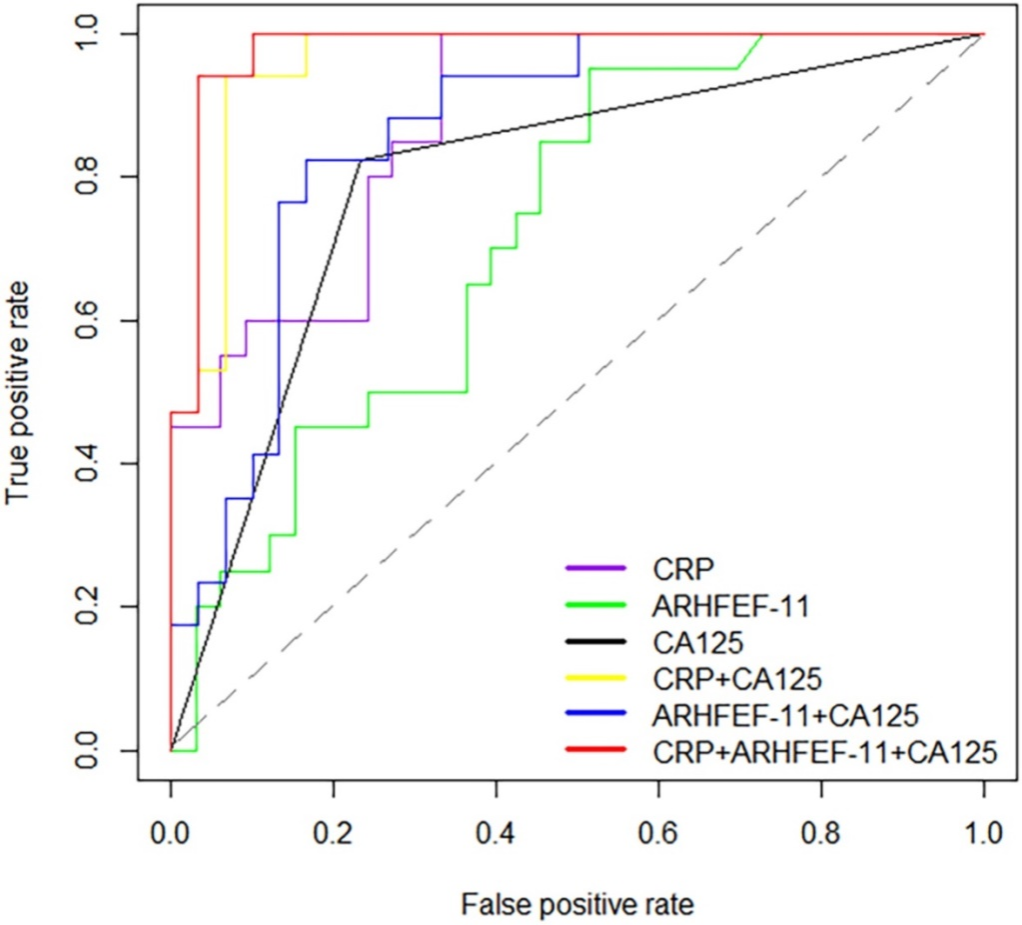
Receiver operator characteristic (ROC) curves for prediction with significant proteins and CA125.

To provide further insight into the utility of these markers, AUC values, sensitivity, specificity and the correct prediction rate were utilized to assess the predictive accuracy of proteins between cancer patients and controls. This analysis showed that CRP had high prediction accuracy in the discrimination of cancer and non-cancer patients with AUC values of 0.88 (sensitivity=1, specificity=0.67). ARHGEF 11 had an AUC of 0.72 significantly different from cancer patient and non-cancer patients. Alone these markers were not considered significantly discriminate this two groups. Therefore, using CRP in combination with CA125 (>35 U/mL for EOC positive) and ARHGEF 11 in combination with CA125 improved the predictability of CA125 alone, especially, improved the correct prediction rate. Expected, combination of CRP, ARHGEF 11 and CA125 improved the predictive performance with an AUC value of 0.98 (sensitivity=0.94, specificity=0.97, Cancer correct prediction rate=16/17, Non-cancer correct prediction rate=29/30) (Table 3 & Fig. 3).

## Discussion

Increased serum CA125 levels are an important indicator for ovarian cancer. However, CA125 levels are not cancer-specific. Ovarian cancer, benign gynecological tumors and other cancers can cause elevation of serum CA125 levels. It is well known that proteins reflect the abnormal phenotype of pathological state and proteomics is one of the power tool to monitor and detect the changes in protein expression. In current study, iTRAQ technology was utilized to perform ovarian cancer plasma proteomics analysis, four proteins were identified as differential expressed proteins for the progression of ovarian cancer after controlling clinical confounding factors (age, menopausal state and admission date). In addition, an independent cohort analysis validated that CRP and ARHGEF 11 were proteins related to ovarian cancer disease progression. Combination of CRP, ARHGEF 11 and CA125 improved the predictability of CA125 alone in the distinguishment of cancer from non-cancer patients significantly, especially improved the specificity, which improved the deficiency of CA125 in the disease diagnosis.

Pathological damage of human organs could lead to the changes of the quality and quantity of plasma proteins, the analysis of plasma proteins is of great significance to the diagnosis of disease and the monitoring of the progression of cancers. Secretory proteins are secreted by histiocytic cells exocytosis into the blood vessels and circulate. Current evidence presents different concentration tendency between the tissue and blood. Simon et al. Shows high levels of B7-H4 protein were detected in ovarian cancer tissue, while low level in all serum samples 22. Welsh et al. reveals significant elevated levels of secretory protein MIC-1 in cancer tissues, as well as highly elevated levels in serum of patients with metastatic prostate, breast, and colorectal carcinomas 23, 24. Our data suggest that up-regulation of secreted protein CRP in ovarian cancer tissues and plasma with the progression of cancers. A comprehensive and systematic analysis the level of CRP would provide a platform in monitoring of the progression of ovarian cancer.

CRP is a plasma protein of hepatic origin, which is a fairly sensitive marker of acute-phase inflammation 25. The common conditions associated with elevations of CRP levels are bacterial infection, inflammatory diseases, cancer, tissue necrosis and trauma 26-28. Previous studies revealed that elevated plasma CRP levels were associated with increased risk of breast cancer, lung cancer, prostate cancer and colorectal cancer 29-31. Trautner et al. also found that elevated CRP levels are indicators of tumor recurrence and poor prognosis 32. Agnoli et al. showed that high CRP was significantly associated with increased breast cancer risk among postmenopausal women 33 and Petekkaya et al. showed that breast cancer patients with a higher serum CRP had shorter survival time compared with normal patients 34. In current study, we observed a significant positive correlation between plasma CRP levels and the occurrence of tumor, but we failed to demonstrate a statistically significant difference in CRP levels between ovarian cancer patients with early stage and late stage, while CRP levels tended to be higher in patients with advanced cancer patients. The same change tendency was observed in ovarian cancer tissue proteomics analysis. The underlying mechanism of the relationship between CRP and cancers are that tumor growth can cause tissue inflammation, which make cancerous cells secrete interleukin 6 (IL-6) and stimulate CRP production in liver 35-37. The results indicated that plasma CRP was an indicator for monitoring the progression of ovarian cancer.

Rho guanine nucleotide exchange factor 11 (ARHGEF 11) is also called PDZ-RhoGEF 38, which is highly expressed in the brain 39, 40. Mizuki et al. found that ARHGEF 11 variants are associated with a higher risk for the onset of schizophrenia in Japanese and further explored the distribution, binding, and functions of ARHGEF 11 in the dendritic spine of the rat cerebral cortex 41. It is also discovered that ARHGEF 11 associated with the risk for type 2 diabetes mellitu 42, 43. There were no studies have revealed the relationship between ARHGEF 11 and cancer directly by far, it might be caused by the dysregulated in RhoGTPase signaling at epithelial tight junctions 44.

## Conclusion

In summary, we performed a plasma proteomics study for ovarian cancer patients and controls. Our results indicate that plasma CRP levels help monitor the progression of ovarian tumor and combination of novel protein biomarkers have a good distinguishment between cancer and non-cancer patients. This study has some limitations. Due to the small sample size of validation set, we failed to validate a statistically significant difference in CRP levels between ovarian cancer patients with early stage and late stage. CRP is also a clinical indicator of inflammation, but our samples did not detect the CRP index in clinical practice, so we could not validate our results from the point of clinic. In future study, additional studies are required to further validate their performance as biomarkers.

## Supplementary Material

Supplementary figures and tables.

## Figures and Tables

**Table 1:** Patient characteristics in the discovery and validation cohorts

Discovery set for iTRAQ quantitative proteomic analysis				
	UF	BOT	Early stage	Late stage
N	24	23	16	17
Age	46.48(32.00-60.11)	46.20(30.65-66.16)	47.39(23.62-57.83)	47.77(38.83-65.04)
Menopause (pre/post)	19/5	18/5	12/4	11/6
CA125	17.92(9.57-39.25)	26.36(6.79-204.10)	62.88(9.70-1600.00)	489.15(41.29-2894.00)
**Validation set for Elisa analysis**				
N	16	17	9	11
Age	46.98(40.13-61.16)	45.40(39.78-68.11)	48.16(39.48-58.28)	47.39(36.83-63.11)
Menopause (pre/post)	12/4	12/5	6/3	7/4
CA125	16.49(10.24-59.37)	18.57(8.25-304.70)	44.73(12.70-308.90)	462.0(50.37-1789.00)

**Table 2:** Differentially expressed proteins identified among four groups.

Gene symbol	Protein name	Ratio BOT/UF	Ratio Early stage/UF	Ratio Early stage /BOT	Ratio Late stage/UF	Ratio Late stage/BOT	Ratio Late stage/Early stage	No. of peptides
CRP	C-reactive protein	1.250	**3.879**	**3.035**	**9.480**	**6.963**	2.509	2
ARHGEF11	Rho guanine nucleotide exchange factor 11	0.653	0.917	1.654	**2.372**	**5.758**	3.530	2
SAA1	Serum amyloid A-1 protein	0.983	**2.543**	**2.498**	**3.581**	**3.650**	1.439	4
SAA2	Serum amyloid A-2 protein	0.871	**2.335**	**3.046**	**2.350**	**3.015**	1.498	4

The ratio was expressed by the average of four repetitions

**Table 3:** Predictive performance of significant proteins in the discrimination of cancers and controls

	Index	AUC	95% Confidence interval	*P* values	Sensitivity	Specificity	Cancer correct prediction rate (correct predictive sample/sample size)	Non-cancer correct prediction rate (correct predictive sample/sample size)
Cancers vs. controls	CRP	0.88	0.79-0.97	<0.001	1	0.67	20/20	22/33
	ARHGEF 11	0.72	0.59-0.86	0.007	0.95	0.48	19/20	15/33
	CA125	0.80	0.66-0.93	<0.001	0.82	0.77	15/20	21/33
	CRP+CA125	0.96	0.91-1.00	<0.001	0.94	0.93	16/17	28/30
	ARHGEF 11+CA125	0.86	0.76-0.97	<0.001	0.82	0.83	14/17	23/30
	CRP+ARHGEF 11+CA125	0.98	0.94-1.00	<0.001	0.94	0.97	16/17	29/30

## Abbreviations

BOT: benign ovarian tumor
UF: uterine fibroid
EOC: Epithelial ovarian cancer
CA125: serum carbohydrate antigen 125
iTRAQ: isobaric tag for relative and absolute quantitation
SCX: Strong Cation Exchange
pAGC: predictive Automatic Gain Control
AUC: Area under the receiver operating characteristic
CPTAC: Clinical Proteomic Tumor Analysis Consortium
RPLC: reverse-phase liquid chromatography
ARHGEF 11: Rho guanine nucleotide exchange factor 11
