# Supplementary material for: Identifying Potential Markers for Monitoring Progression to Ovarian Cancer Using Plasma Label-free Proteomics
Source: J Cancer. 2021 Jan 15;12(6):1651–9. doi: 10.7150/jca.50733 (PMC7890305; doi:10.7150/jca.50733)

Table S1 The details of the plasma and tissue proteins

PLASMA-UP & CPTAC-UP	PLASMA-UP & CPTAC-DOWN	PLASMA-DOWN & CPTAC-UP	PLASMA-DOWN & CPTAC-DOWN
C4BPB	PIGR	PIP4K2A	SELL
CRTAC1	MYH13	ZMYM4	PROZ
PKP3	CNDP1	FIP1L1	APOA2
CDC5L	COLEC11	FOXRED1	PON3
EFEMP1	VTN	THRAP3	SERPINF2
CADPS	HRG	UBTF	ALB
DSP	NEB	C1RL	APOC1
KIAA0195	CPB2	ADAR	APOA1
HDAC5	MFI2	KIF13A	APOA4
RCN1	OAF	PTPRJ	PLG
F13B	SERPINA10	RPL5	SERPINC1
C1orf122	C4B	SPARCL1	TF
WDR7	ABCB9	GEMIN6	REEP6
SAA2	APOD	PSMG1	ITIH2
COPB1	C7	BAG6	CLEC3B
UBR1	CP	ALDOA	PON1
ATM	F9	VPRBP	TTR
MGA	C9	PPP1CA	AHSG
JTB	SERPIND1	APOB	AFM
MAEA	C5	IL1RAP	ITIH1
QKI	TGFBI	SPECC1L	APOE
VWF	A1BG	COPB2	C4A
LAD1	C8B	DMD	VPS54
SSFA2	C8A	MKL2	CRISP3
CEP350	C3	PPP1R9B	IGFALS
FKBP4	CPN1	DENND1B	F7
EYA2	HPX	RMND1	SHBG
MYO5B	AZGP1	SOD3	GC
H3F3B	CFH	ZFAND3	ITIH4
CTAGE1	CPN2	SCYL2	SERPING1
RSF1	AMBP	MCAM	SERPINA4
UPF1	LGALS3BP	CUL7	FAM208B
PIK3R1	ADAMTS1	EXOC3	KNG1
MINPP1	CFHR1	GSN	LCAT
SUMO3	GOLGB1	INTS6	IGJ
PRG4	GNPTG	SMC3	F2
PLEKHG2	LRG1	ABR	HABP2
TRAPPC5	FCN2	CGN	F10
RIC8A	CFHR2	DOPEY2	RBP4
PRDX2	NTPCR	BLMH	CCBL2
EEA1	ITIH3	GPX5	KLKB1
KLHL7	CFHR5	RNF213	CFI
TGFB1	LAMA1	DYSF	APOH
HEPH	AGT	F13A1	GPX3
ADIPOQ	SERPINA1	SIK2	CFP
ARHGEF11	C8G	INADL	PGLYRP2
VCL	IGLL5	BRPF1	SERPINA3
IGFBP2	B2M	GPLD1	C6
CDC45	ORM2	PRUNE	C2
PF4	GNPAT	PRKAG2	ICAM2
KIAA1524	CRYZ	IGF2	SERPINA6
CDH12	C1QA	L1CAM	TIAM1

PLASMA-UP & CPTAC-UP	PLASMA-UP & CPTAC-DOWN	PLASMA-DOWN & CPTAC-UP	PLASMA-DOWN & CPTAC-DOWN
VCAM1	NFASC	LUM	SEPP1
HERC1	ZFC3H1	RARRES2	C1QC
PPBP	FCN3	NCAM1	APOF
MGP	MST1	FBLN1	APOM
VIM	FETUB	GFPT2	A2M
COMP	TBC1D2B	USP42	APOL1
LIMA1	ITGAL	SPC25	CDH5
BBS9	SZT2	CRTAP	HGFAC
THBS1	PTGDS	KIAA1211	BTD
INPP5F	PZP	CRADD	SLC35A2
CRP	LBP	LDHB	LGR5
PTAR1	PROS1	LPAR2	SERPINA5
GJA1	F5	FN1	GTPBP2
AOC3	QSOX1	APOC4	F11
KIF18A	CFHR4	DZIP3	USP53
ICOSLG	ORM1	BCHE	IGFBP3
ZNF770	S100A9	ADCY8	FGA
	C1QB	ZKSCAN5	NCK1
	F12	LYVE1	ICE1
	FGB	NEURL4	ECM1
	HSPA4L	HBD	MMRN2
	CD5L	HBB	FGG
	CHL1		UBN2
	ICAM1		SPCS2
	STAT1		APOC3
	GGH		CNOT6L
	PTPN14		DDX47
	PSD4		APOC4-APOC2
	THOC7		CST3
	FCGBP		IGFBP4
	SERPINA7		PROC
	TTN		MAOB
	LCP1		COBL
	FBN2		USP35
	HP		ZZEF1
	EVL		INTS4
	PRPF18		SRP54
	CCAR1		BGN
	ZNF740		C1S
	C4BPA		CD14
	AASDHPPT		ATRN
	CDH13		ANK3
	H6PD		ZNF346
	CNST		TBC1D8B
	CFD		APMAP
	SIN3A		HPR
	DDX60		ATP11C
	AP3B2		HSPA5
	PVR		MYO1E
	PNN		CAMP
	LPA		CD44
	CLEC16A		KIFC1
	VASN		PNPLA8
	CTCFL		SAE1
	CEP85		SERPINF1

PLASMA-UP & CPTAC-UP	PLASMA-UP & CPTAC-DOWN	PLASMA-DOWN & CPTAC-UP	PLASMA-DOWN & CPTAC-DOWN
	SAA1		PCNT
	MYO1D		RASAL2
	CLTC		PRPF4B
	EIF3C		SARAF
	VAC14		VPS53
	MED13L		STRA6
	MATN4		REPIN1
	DOCK5		NEK1
	GALE		PCYOX1
			INTS1
			C1R
			ZFYVE16
			PLA2G15
			NRDE2
			TPT1
			TRAF7
			PPP2R5C
			TIAL1
			GOLGA4
			BICD1
			MED15

Figure S1. Overlapped proteins between tissue and plasma proteomics analysis. (a) tissue proteomics analysis; (b) plasma proteomics analysis.

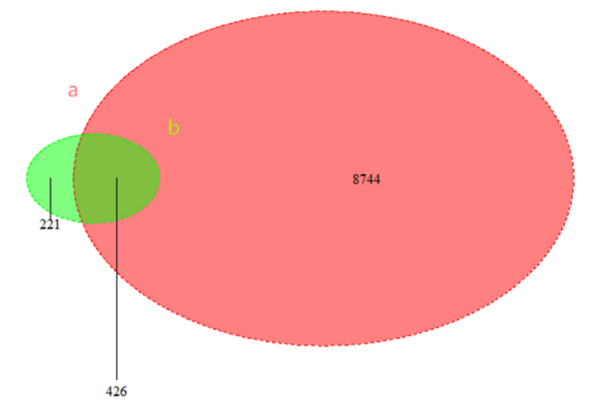

Supplement: Supplementary file 1 — Supplementary figures and tables. [file jcav12p1651s1.pdf]
